# A lactate dehydrogenase ELISA-based assay for the *in vitro* determination of *Plasmodium berghei* sensitivity to anti-malarial drugs

**DOI:** 10.1186/1475-2875-11-366

**Published:** 2012-11-05

**Authors:** Pamela Orjuela-Sánchez, Erika Duggan, John Nolan, John A Frangos, Leonardo JM Carvalho

**Affiliations:** 1 La Jolla Bioengineering Institute, 3535 General Atomics Court, Suite 210, San Diego, California (CA), 92121, USA; 2 Laboratory of Malaria Research, Oswaldo Cruz Institute, Fiocruz, 21040-900, Rio de Janeiro, Brazil

**Keywords:** *Plasmodium berghei*, *In vitro* culture, schizont, synchronized infection, drug *in vitro* assay, anti-malarials, IC_50_ values, *lactate dehydrogenase*, ELISA

## Abstract

**Background:**

*Plasmodium berghei* rodent malaria is a well-known model for the investigation of anti-malarial drug efficacy *in vivo*. However, the availability of drug *in vitro* assays in *P. berghei* is reduced when compared with the spectrum of techniques existing for *Plasmodium falciparum*. New alternatives to the current manual or automated methods described for *P. berghei* are attractive. The present study reports a new ELISA drug *in vitro* assay for *P. berghei* using two monoclonal antibodies against the parasite lactate dehydrogenase (pLDH).

**Methods:**

This procedure includes a short-*in vitro* culture, the purification of schizonts and the further generation of synchronized mice infections. Early stages of the parasite are then incubated against different concentrations of anti-malarial drugs using micro-plates. The novelty of this procedure in *P. berghei* relies on the quantification of the drug activity derived from the amount of pLDH estimated by an ELISA assay using two monoclonal antibodies: 14C1 and 19G7. The IC_50_s obtained through the ELISA assay were compared with those from the micro-test.

**Results:**

The initial parameters of the synchronized samples used in the *in vitro* assays were a parasitaemia of 0.5% and haematocrit of 1%, with an incubation period of 22 hours at 36.5°C. pLDH detection using a 14C1 coating at 10 μg/ml and 19G7 at 2.5 × 10^-3^ μg/ml provided good readouts of optical densities with low background in negative controls and specific detection levels for all parasite stages. IC_50_s values derived from the ELISA assay for artesunate, chloroquine, amodiaquine and quinine were: 15, 7, 2, and 144 nM, respectively. When artesunate and chloroquine IC_50_s were evaluated using the micro-test similar values were obtained.

**Conclusion:**

This ELISA-based *in vitro* drug assay is easy to implement, fast, and avoids the use radioisotopes or expensive equipment. The utility of this simple assay for screening anti-malarial drug activity against *P. berghei in vitro* is demonstrated.

## Background

Murine malaria models have contributed to the understanding of the biology and pathology of human malarias. Although the immune system and drug pharmacokinetics between humans and rodents may be different, the drug sensitivity profile between these *Plasmodium* species is mostly shared. Drug efficacy studies in small rodents have been mainly performed *in vivo* using different strains of *Plasmodium berghei* and the classical four-day suppressive test [1]. The use and development of *in vitro* techniques for anti-malarial drug-screening in *P. berghei* has been less prominent, especially when compared with the variety of *in vitro* assays described for *Plasmodium falciparum*. *In vitro* assays are complementary to *in vivo* tests, and in *P. berghei* some advantages of the *in vitro* assays include: i) the measurement of intrinsic anti-malarial activity, excluding confounding factors from the host such as the immune system; ii) the evaluation of larger number of compounds in one experiment with possible prediction of an *in vivo* outcome, and iii) the identification of discrepancies in the sensitivity profiles of *P. berghei* and *P. falciparum*.

Different drug *in vitro* assays have been adapted and developed for *P. berghei*. These assays include: the WHO schizont maturation test (micro-test) [2], the radioisotopic assay [3] and the measurement of parasite nucleic acids using fluorescent dyes and flow cytometry [4,5]. More recently, a luminescence assay using a transgenic line of *P. berghei* expressing luciferase was also developed [6]. These techniques can be limited by labour-intensive steps, disposal of radioactive waste and high costs.

Early studies of the metabolic pathways of *Plasmodium* led to the identification of enzymes structurally different from the host, such as lactate dehydrogenase (LDH) [7]. *Plasmodium* parasites require intense LDH activity to ensure the high metabolism of carbohydrates during the complex intraerythrocytic cycle [8]. These singularities pointed to parasite LDH (pLDH) detection as a good target for the development of colorimetric [9] and ELISA [10] based drug *in vitro* assays for *P. falciparum*. The aim of this study was to develop an *in vitro* enzyme-linked immunosorbent drug assay for *P. berghei*, using two monoclonal antibodies against pLDH and the semi-automated micro-dilution technique [11]. This *in vitro* susceptibility test starts with *P. berghei* mouse infections followed by a short *in vitro* culture and a synchronization procedure to generate the *P. berghei* samples to be evaluated in the drug *in vitro* assays. The measurement of drug activity is derived from the relative amount of *P. berghei* LDH (estimated by ELISA) as a function of the drug concentration.

## Methods

### Short-term parasite *in vitro* culture and synchronization

13-week old C57BL/6 (The Jackson Laboratory, Sacramento, CA) mice were infected intraperitoneally (IP) with 1 × 10^3^*P. berghei* ANKA (PbA) or *P. berghei* ANKA expressing GFP (PbAGFP) [12] (a donation from the Malaria Research and Reference Reagent Resource Center (MR4), Manassas, VA; deposited by MF Wiser and M Hollingdale and CJ Janse and AP Waters, respectively; MR4 number: MRA-671 and MRA-865). At a parasitaemia of 2 - 3%, mice were anesthetized (ketamine, 150 mg/kg and xylazine, 10 mg/kg) and 0.5 ml of blood was collected by retro-orbital puncture, after which mice were euthanized by an IP injection of Euthasol (100mg/kg). Animal handling and care followed the NIH Guide for Care and Use of Laboratory Animals. All protocols were approved by the La Jolla Bioengineering Institutional Animal Care and Use Committee.

Infected heparinized blood samples were washed twice in phosphate-buffered saline (PBS) solution and cultured in media RPMI 1640 (Gibco, Life Technologies) supplemented with 20% inactivated fetal bovine serum (complete RMPI) following the procedures described by Janse and Waters [13]. Cultures were flushed with a standard gas mixture of 5% O_2_, 5% CO_2_, 90% N_2_ and incubated for 22 hours at 36.5°C. Purification of fully mature schizonts from uninfected red blood cells or younger parasites’ stages was performed following the protocol described by Janse *et al.*[14] with some modifications. Briefly, 25 ml of the overnight parasite culture was transferred to a 50 ml falcon tube (Becton, Dickinson) and 8 ml of 55% Nycodenz (v/v, PBS) was added to the bottom of the tube. Tubes were centrifuged at 450 g in a swing-out rotor for 30 minutes without brake. After centrifugation, the visible suspended brown ring containing mostly mature schizonts was carefully collected into another falcon tube. Schizonts were washed in complete RPMI media, resuspended in 100 μl of PBS and injected intravenously (IV) into the pre-warmed tails of 13-week old C57BL/6 mice. Between 48 to 72 hours post-infection, an optimal synchronized parasitaemia of 1 to 3% was reached. Mice were anesthetized as previously described and parasite blood samples were collected. To adjust the parasitaemia and haematocrit for the *in vitro* assay samples, a pool of blood from uninfected mice was also gathered.

Mice parasitaemia and synchronized infections of *P. berghei* were analysed by flow cytometry and Hemacolor (Merck) stained thin blood smears. Tail blood samples from infected and uninfected mice were diluted 100-times in PBS, and subsequently acquired in a FACSCalibur cytometer (Becton Dickinson). Samples were excited using a 488 nm argon laser and GFP emission was detected with a 530/30 nm band pass filter. FCS Express (De novo Software) was used for all flow cytometry analyses by first gating for intact erythrocytes by side scatter and forward scatter parameters, and subsequently determining the proportion of GFP positive cells. The fluorescence intensity and the forward-scattered light of at least 10,000 cells per sample were measured. A negative control sample from an uninfected mouse was tested in parallel to define the threshold of positivity for the parasitaemia.

### Drug *in vitro* assays

The anti-malarial drugs evaluated in the *in vitro* assays were: chloroquine diphosphate salt, amodiaquine dihydrochloride dihydrate, quinine hydrochloride dihydrate and artesunate (all from Sigma-Aldrich). The drugs were diluted in ethanol 70% (v/v, ultrapure water) with the exception of chloroquine that was diluted in ultrapure water. The stock solutions of each drug were used to prepare two-fold dilution series in complete RPMI medium. The dilutions ranging from: 3.8-240 nM chloroquine, 1–65 nM amodiaquine, 50–3150 nM quinine-HCL and 0.6-40 nM artesunate were distributed (25 μl per well) in 96-well plates (Corning, Life Science) where the drug *in vitro* assays were performed.

Uninfected and infected red blood cells containing primarily early trophozoites were washed twice in PBS prior to use in the *in vitro* assays. Infected red blood cells were diluted in complete RPMI medium to a final parasitaemia of 0.5% and 1% of haematocrit and dispensed (225 μl per well) into the pre-dose drug plates. In each experiment, wells with uninfected and infected red blood cells without drug were included as negative and positive controls of growth, respectively. In addition, aliquots from the positive controls were frozen immediately in order to assess pLDH activity at time zero of incubation. The drug *in vitro* plates were incubated in gas atmosphere of 5% CO_2_, 5% O_2_, and 90% N_2_ at 36.5°C for 22 to 24 hours. At the end of the incubation period, thin blood smears of positive controls were performed to confirm the presence of mature schizonts (with 8 to 16 merozoites).

The drug concentrations that inhibited 50% of parasite growth (measured by optical densities – ODs in the ELISA assays) compared to the control samples without drug (IC_50_s) were calculated using HN-NonLin [15]. Drug-response curves were plotted in GraphPad Prism. Data were expressed as mean ± standard deviation (SD), unless otherwise indicated.

### Enzyme-linked immunosorbent assay

Monoclonal antibodies 14C1 (a kind donation of Dr. Michael Makler) and 19G7 (purchased from Flow Inc., Portland, OR) developed for pLDH-based malaria diagnostic tests [16] were used as capture and detection antibodies, respectively (these monoclonal antibodies are now owned by AccessBio, Somerset, NJ). Biotinylation of 19G7 (10 μg/ml) was carried out using the EZ-Link Sulfo-NHS-LC-Biotinylation Kit (Pierce Biotechnology) following the standard protocols provided by the manufacturer. The 2-(4-hydroxyazobenzene) benzoic acid (HABA) assay also included in the EZ-Link Sulfo-NHS-LC-Biotinylation Kit was used for measuring the level of biotin incorporation per immunoglobulin molecule.

The ELISA technique described above was standardized from the DELI assay developed for *P. falciparum* with modifications [10]. Nunc MaxiSorp flat-bottom 96 well plates (Gibco) were coated with 100 μl of capture antibody 14C1 (10 μg/ml) and incubated at 4°C for 24 hours. Plates were washed with phosphate-buffered saline (PBS) 0.025% (v/v) Tween 20 (PBS/Tween) and blocked with 1% (w/v) Bovine Serum Albumin Fraction V (Roche) (PBS/BSA). After 24 hours of incubation at 4°C, the plates were washed, covered with plastic seals, refrigerated and used within a month.

Once the drug *in vitro* assays were performed, the plates were frozen/thawed three-times and the haemolyzed samples were homogenized. 100 μl of haemolyzed samples, undiluted and diluted (1:10, 1:50 and 1:100 in PBS), was transferred into the coated ELISA plates and incubated for 1 hour at 37°C. The plates were washed and 100 μl of a 1:4,000 dilution (2.5 × 10^-3^ μg/ml) of 19G7 biotinylated antibody was added. After 1 hour of incubation at 37°C, plates were washed and incubated for 30 minutes with 100 μl of a 1:10,000 dilution (0.125 μg/ml) of peroxidase-conjugated streptavidin preparation (1.25 mg/ml) (Pierce ). After the last wash, 100 μl of TMB (3,3´,5,5´-tetramentylbenzidine) (1-Step Ultra TMB-ELISA, Thermo Scientific) was added to the plate and incubated for 20 minutes at room temperature. The reaction was stopped by adding 100 μl of 2 M H_2_SO_4_ solution, and read at 450 nm in a μQuant micro-plate spectrophotometer (Bio-Tek Instruments).

## Results and discussion

Initial ELISA assays aimed to evaluate the antibody recognition capability of PbAGFP samples and establish the optimal antibody concentrations to be used. ELISA assays were first conducted in uncultured and asynchronous parasites adjusted to 1% and 2.5% of parasitaemia and haematocrit, respectively. Initial coating plates concentrations (10, 50 and 100 μg/ml) of primary antibody (14C1) versus the secondary antibody (19G7) (1:1,000 [0.01 μg/ml] to 1:64,000 [1.56 × 10^-4^ μg/ml] two-fold dilution) were tested. These preliminary experiments showed that independently from the coating concentration used, dilutions of the secondary antibody below 1:6,000 (1.67 × 10^-3^ μg/ml) did not significantly differ in their antigen detection capability. Using a concentration of 10 μg/ml of 14C1 and a 1:4,000 dilution (2.5 × 10^-3^ μg/ml) of 19G7, the assay generated absorbance values of 2.7 (± 0.01) for infected samples and 1.2 (± 0.01) for negative controls: uninfected blood and complete RPMI media. Based on these preliminary results, next assays attempted to reduce the high absorbance values obtained for the negative controls. Simple modifications were performed: (i) reduced the haematocrit of the samples to 1%, (ii) supplemented the PBS used for washes with Tween-20 0.05%, (iii) and performed and extra wash at all ELISA steps (four in total). These basic protocol changes significantly reduced the absorbance from the negative controls from 1.2 (± 0.01) to 0.2 (± 0.02) whereas the infected samples still showed good detection levels (1.5 ± 0.3).

**Figure 1:**
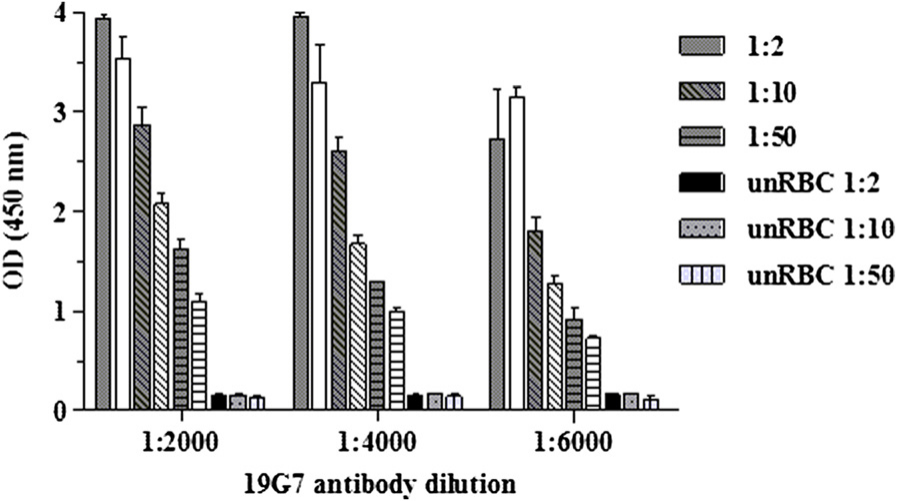
**Effect of sample dilution in pLDH detection – ELISA standardization.** Vertical bars represent mean optical density (OD) values ± SD obtained from PbAGFP cultured schizonts at 0.5% or 1 % of parasitaemia and uninfected red blood cells (unRBC) at 1% of haematocrit. Grey and white bars represent schizonts culture at 1% and 0.5% of parasitaemia, respectively. Three different concentrations of 19G7 (secondary antibody) were tested (X-axis). Mean is derived from three independent assays.

Parasite blood samples used in these preliminary tests were uncultured and asynchronous with different stages of the parasite producing different levels of pLDH. Since the *in vitro* drug method proposed here is based on the schizont maturation inhibition assay [2], next experiments were directed to evaluate the ELISA standardized conditions in late stages of PbAGFP. The *P. berghei* life cycle takes approximately 24 hours and under static *in vitro* culture conditions the asynchronous parasites from the mice infection develop into mature schizonts that do not burst in culture. Two different parasitaemias (0.5% and 1%) of cultured mature schizonts obtained after 22 hours of *in vitro* culture were evaluated in the ELISA assays. Since the absorbance expected for these stages was the highest [17], three different dilutions (1:2, 1:10 and 1:50 in PBS) of the samples were tested. The performance of the ELISAs using a 0.5% or 1% parasitaemia showed no critical differences and both worked well. At 0.5% parasitaemia, culture dilution 1:10 resulted in absorbance values in the range of 2.2 to 1.3 (1:2,000 [5.0 × 10^-3^ μg/ml] to 1:6,000 [1.67 × 10^-3^ μg/ml] secondary antibody dilution, respectively) (Figure 1). At 1% parasitaemia, both 1:2 and 1:10 culture dilutions resulted in high absorbance values (between 4.0 and 2.6, except at 1:6,000 secondary antibody dilution, absorbance = 1.8). Signal-to-noise ratios at culture dilution 1:50 were not as good as dilution 1:10. It was concluded therefore that in cultures with 0.5% parasitaemia diluted 1:10, any of these 19G7 dilutions were appropriate. Dilution 1:4,000 (2.5 × 10^-3^ μg/ml) of 19G7 was selected for further use in this study as it provided a better signal-to-noise ratio than 1:6,000, and allowed using lower amount of secondary antibody than dilution 1:2,000. For cultures at 1% parasitaemia, the combination of 1:10 culture dilution with 19G7 dilution 1:6,000 would be appropriate as well. The use of different combinations does not seem to be critical for the assay, as long as the absorbance values fall within the non-saturation (non-plateau) range. As can be seen in Figure 1, the plateau is far from being reached at 1:10 dilutions (that is, 1:2 dilutions still provide absorbance values well above those observed with 1:10 dilutions).

**Figure 2:**
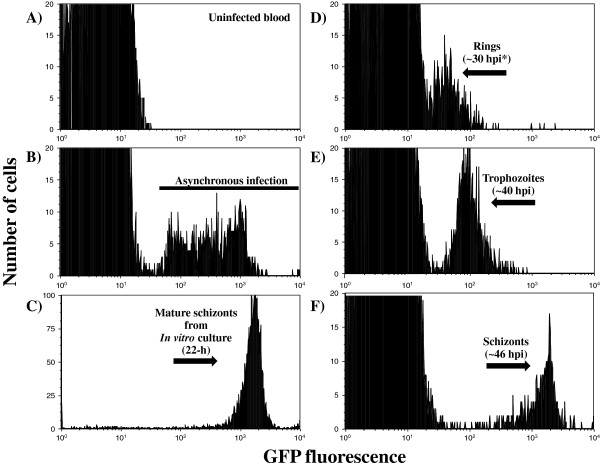
**Flow cytometry analyses of synchronized infections of *P. berghei *in C57BL/6 mice.A)** Panel showing uninfected red blood cells (negative controls). **B)** Asexual blood-stages of PbAGFP from infected mice with normal day-night light regime. **C)** Highly fluorescent mature schizonts purified after 22 hours of *in vitro* culture used to generate synchronized *P. berghei* infections in mice. **D)** Rings, **E)** trophozoites and **F)** schizont-enriched populations from synchronized infections after one cycle of replication in mice. *hpi=hours post-infection.

The development of *P. berghei* in mice is relatively asynchronous and unsuitable for schizont inhibition assays, since most of the anti-malarial drugs are parasite stage-specific. In *P. falciparum*, synchronization can be easily performed by sorbitol treatment [18]. However due to alterations in the permeability of murine erythrocytes this procedure is less efficient in *P. berghei*[19]. A suitable alternative for *P. berghei* synchronization is the development of synchronous infections in mice. Following the procedures described by Janse and Waters [13], synchronized infections of PbA and PbAGFP were generated and followed by thin blood smears and/or flow cytometry (Figure 2). PbAGFP express GFP throughout the whole life cycle and displays different GFP-fluorescence intensity during each stage [12] facilitating the analyses of mice synchronized infections by flow cytometry. As expected, asexual blood stages of PbAGFP with different parasite stages were found during the course of the infection (Figure 2, histogram B). Once the parasites were collected from the mice and cultured overnight, a highly pure and concentrated population of mature schizonts was gathered through the use of the Nycodenz gradients (Figure 2, histogram C). Collected schizonts (approximately 1 × 10^7^) were then injected IV into mice and 30 hours post-infection a highly synchronized parasitaemia of 1-3% was observed (Figure 2, histograms D to F). This procedure allowed the acquisition of the early stage parasite samples required for the conduction of the drug *in vitro* assays. This protocol was also useful for PbA (wild type, not expressing GFP) synchronization but parasite development was only followed by thin blood smears.

**Figure 3:**
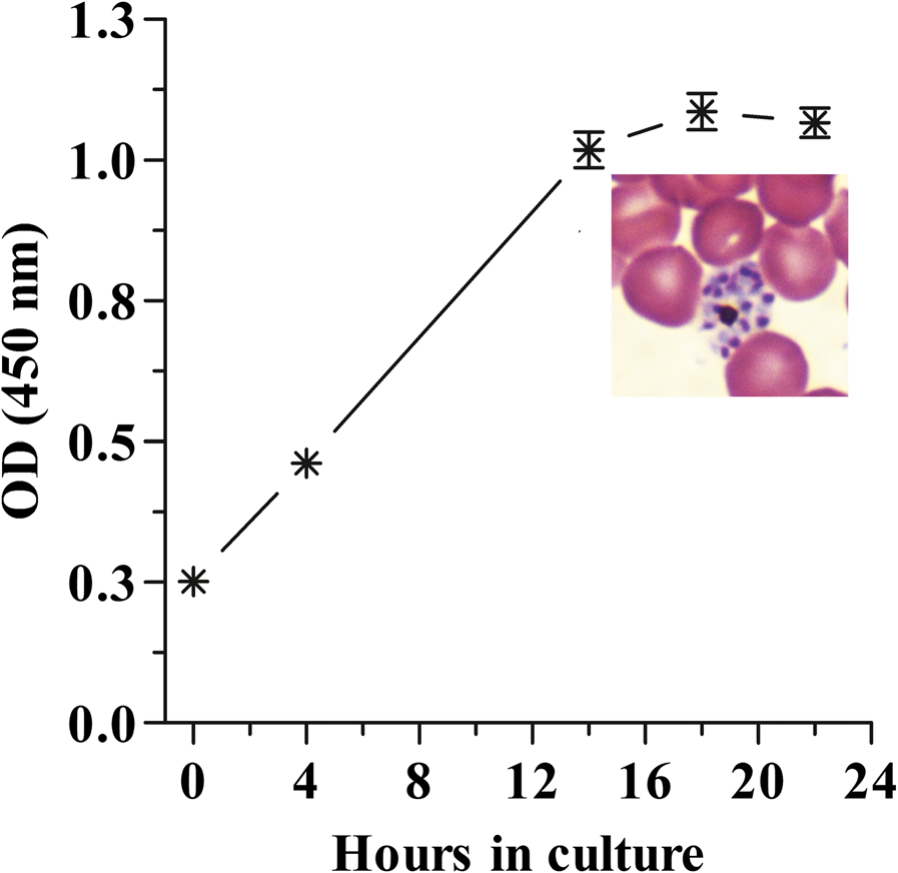
**Relationship between growth, stage development and pLDH optical density of synchronized cultures of *P. berghei.*** Parasites were cultured under drug *in vitro* assay conditions without drug. Samples were collected and frozen at the different time points. Asterisks represent the mean and standard error of two different experiments. A picture of a mature schizont (usually containing 8–16 nuclei) collected at the end of the assay (hour 22) is also shown.

After having standardized the synchronized infections in mice, the next objective was to confirm the capability of the ELISA assay to detect pLDH activity throughout the different stages of the parasite life cycle in samples that were grown under the *in vitro* assay conditions. To do this, parasites from synchronized infections were collected in early stages of development (early trophozoites adjusted to 1% of haematocrit and 0.5% of parasitaemia) and short-cultured in 96-well plates. Incubation of plates was performed at 36.5°C in a desiccator plastic chamber filled with a sterile gas mixture (5% CO_2_, 5% O_2_, and 90% N_2_). Higher incubation temperatures (37°C and 37.5°C) were also tested but under these conditions parasites did not develop into mature schizonts and the presence of degenerated schizonts was common. Over the 22 hours of the incubation period parasite samples were collected at different time points in order to perform thin blood smears and the measurement of pLDH activity. In Figure 3 it can be observed how pLDH levels increased as the parasite grew during the incubation time. Ring samples at time 0 of incubation had an initial OD value of 0.25 (± 0.02). After 4 hours of incubation, intracellular growth occurred, trophozoites predominated in the culture and the OD nearly doubled to 0.45 (± 0.02). Ten hours after the second sample was taken (schizonts) the OD reached 1.0 (± 0.06), with a slight increase at 18 hours. The OD remained stable until the end of the culture (22 hours) when fully mature schizonts with more than 8 nuclei were observed.

These simple growth tests confirmed that the short-culture conditions to be used in this *in vitro* assay supported well the parasite growth and the feasibility of this ELISA-based technique to detect it. These results also show that the maximal pLDH activity was generated when the parasites developed from early trophozoites into schizonts, which is consistent with previous reports in *P. falciparum* where the peak of L-lactate (product of pLDH activity) coincided with the beginning of the schizogony [17,20]. There was no significantly increase in pLDH values after 16 hours of incubation when nuclear division and merozoite formation start to occur. These findings indicate that this assay is appropriate to evaluate drugs that affect the parasite’s growth and maturation but not for drugs targeting DNA synthesis during schizogony. In addition, in the absence of an efficient long-term continuous culture of *P. berghei*[21,22] the drug *in vitro* assay proposed here is based on a single cycle. Therefore, this test is not suitable for the evaluation of drugs that have no apparent effect until division and reinvasion of new red blood cells by the daughter merozoites occur (delayed-death phenomenon described with antibacterial drugs in *P. falciparum*) and whose effect can only be estimated in the next developmental cycles [23]. For this kind of drugs, other *in vitro* assays for *P. berghei* based on DNA synthesis [5] and luciferase schizont-specific expression are available [6]. The recent report of a new *in vitro* culture technique for *P. berghei* that allows reinvasion of normal mouse red blood cells and continuous culture for over two weeks [24] might also overcome this limitation.

**Figure 4:**
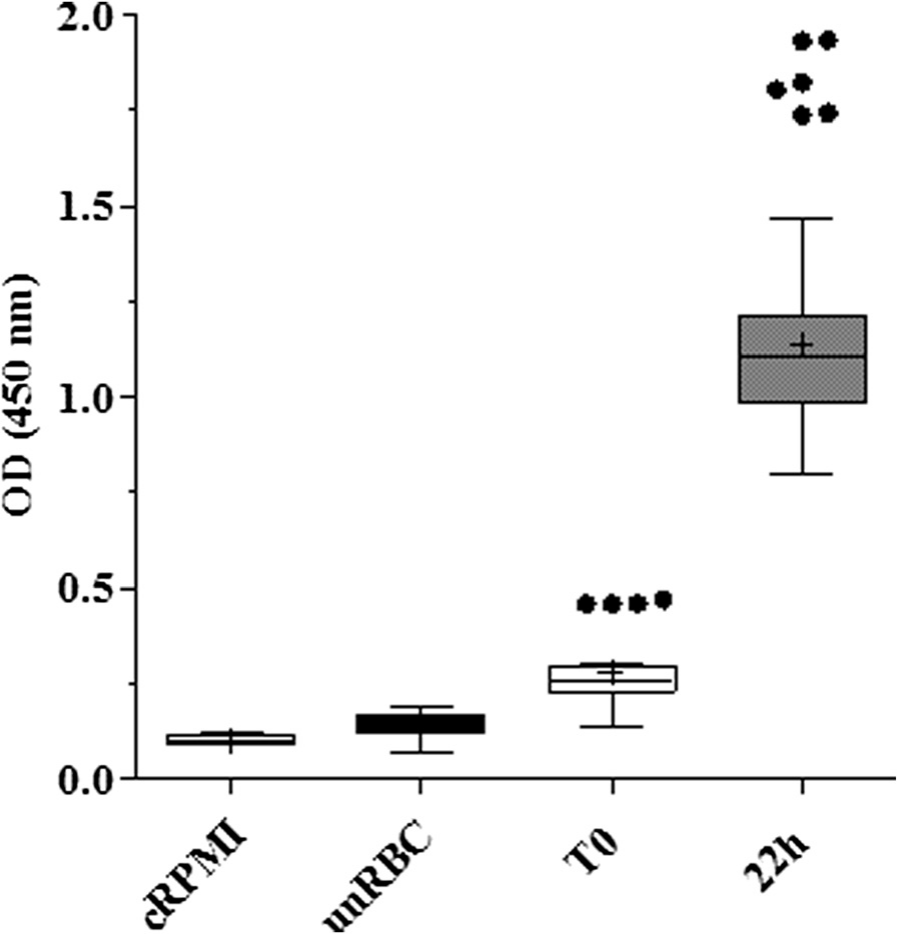
**Optical density readings from drug *in vitro *assay controls.** pLDH OD values for negative controls: complete RMPI media (cRPMI, N = 9 wells), uninfected red blood cells (unRBCs, N = 45 wells) and positive controls (N = 84 wells): infected red blood cells without drug collected at time zero of incubation (T0) and after finished the assay (22h). Wells from different assays were plotted together and at least three independent assays were performed per each group. Values are displayed in box plots, means and outliers are represented by solid crosses and black circles, respectively.

For the *in vitro* assays, early parasite stages (rings) obtained from synchronized infections were used. Parasites were incubated with the different concentrations of the anti-malarial drugs at 36.5°C for 22 hours. Slides confirming the fully maturation of healthy schizonts in the positive control wells were always performed at the end of each assay. Figure 4 shows the absorbance recorded for positive and negative controls during the drug *in vitro* assays. Absorbance values of negative controls were 0.14 (± 0.03) and 0.10 (± 0.01) for uninfected red blood cells and complete RPMI media, respectively. The negative controls showed significantly lower absorbance (Fisher Exact Test, p = 0.0001) than infected samples, regardless of the time of collection. Infected samples at time zero of growth also showed low absorbance values (0.3 ± 0.10), as expected for early stages, but significantly doubled (Fisher Exact Test, p = 0.0001) the OD values of the negative controls. After 22 hours of incubation, the samples from time zero of growth tripled their absorbance values to 1.1 (± 0.2 SD). These results clearly show the suitability of the antibodies to for *P. berghei* detection.

**Figure 5:**
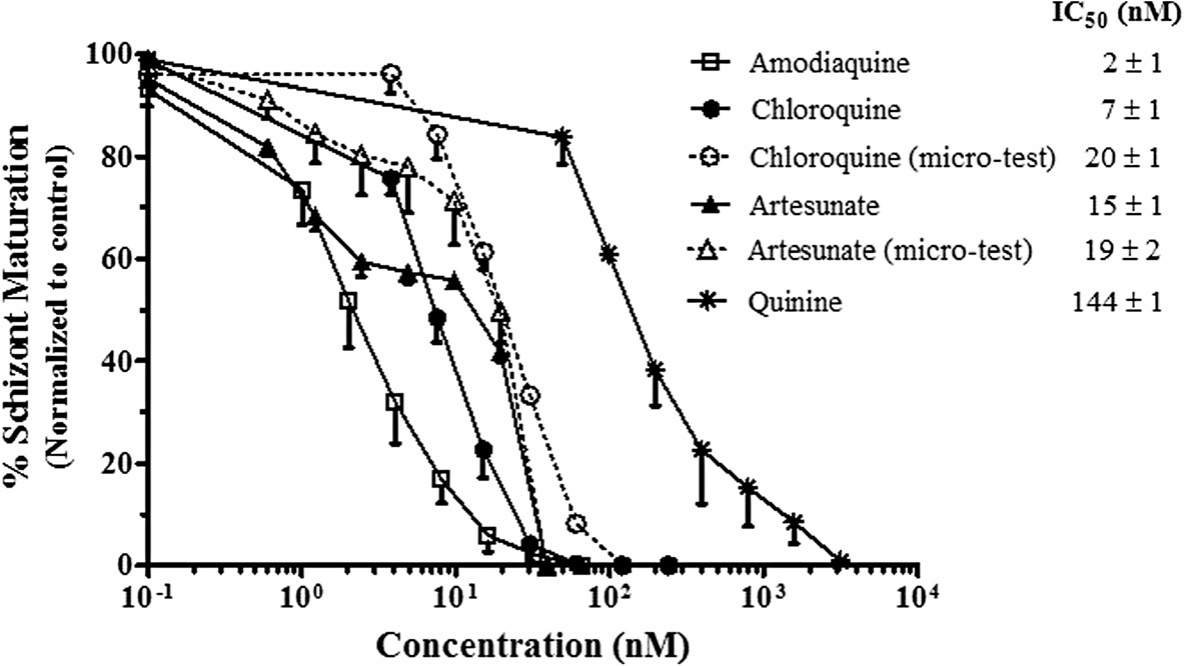
***In vitro *susceptibility of *P. berghei *to different anti-malarial drugs.** Inhibition of schizont maturation by amodiaquine (squares), chloroquine (solid circles), artesunate (solid triangles) and quinine (asterisks). Curves derived from micro-test assays are displayed as dashed lines and open symbols. Each point represents the mean and the standard error of at least three independent assays for PbA and PbAGFP. The IC_50_ values and standard error of the media are shown in the legend.

The *in vitro* response of *P. berghei* to common anti-malarial drugs can be observed in Figure 5. Since no differences between the IC_50_s values of the wild type (PbA) and the transgenic line (PbAGFP) were observed, curves for both parasites were plotted together. The IC_50_ of chloroquine and artesunate was also investigated for PbAGFP using the micro-test [2] with similar results for both techniques (Figure 5). The IC_50_ values for artesunate obtained in this work (15 nM) are in accordance with previous reports for this species [5,6]. Janse *et al.*[5] developed and *in vitro* assay for *P. berghei* analyzing the amount of parasite DNA through Hoechst staining and flow cytometry and reported IC_50_ values for artesunate of 11 nM. More recently, the same group developed another anti-malarial drug screening *in vitro* assay using a transgenic line of *P. berghei* expressing luciferase [6]. Through this highly sensitive technique, the recorded IC_50_ values of artesunate ranged from 4 to 26 nM. IC_50_ values for chloroquine in sensitive *P. berghei* strains have also been described through the use of the different techniques. Early reports of IC_50_s for chloroquine sensitive lines (NK65 strain) using the radioisotopic assay ranged between 155 nM [25] and 230 nM [3]. A report in another murine *Plasmodium* species *Plasmodium chabaudi* described an IC_50_ value of chloroquine of 30 nM using the radioisotopic technique [26]. More recently, IC_50_ values of 30 nM were also described in *P. berghei* using the luciferase assay [6]. In the present study, the IC_50_s values for chloroquine sensitive lines (PbA and PbAGFP) were 7 nM and 20 nM using the pLDH ELISA and the micro-test, respectively (Figure 5); both values are within the range of IC_50_ values described for sensitive chloroquine strains of *Plasmodium* in rodents [3,6,25,26] and similar to those reported in *P. falciparum*[10,27-30] and *Plasmodium vivax*[24,31]. The reports of IC_50_s for amodiaquine and quinine in *P. berghei* are more restricted, mainly derived from the radioisotopic assays: 430 nM and 1500 nM, respectively [3]; another study showed an IC_50_ of 210 nM for quinine-HCL using again the radioisotopic technique [25]. In this work was found that *P. berghei* ANKA lines were sensitive to amodiaquine (IC_50_ of 2nM) and quinine (IC_50_ of 144 nM). These values have been also described in sensitive strains of *P. falciparum*[10,27-30] and *P. vivax*[32]. However, comparison of *in vitro* IC_50_ data among different *Plasmodium* species should be cautiously made and was not the purpose of the present study. Comparisons of these results, with previous reports in *P. berghei* can be also limited since available IC_50_s have been obtained through different techniques and conducted with different strains, hosts, drug dilution protocols and under diverse parameters of parasitaemia and haematocrit that can influence the IC_50_ results [27]; moreover early *in vitro* assays described in *P. berghei* were usually conducted without synchronization procedures [3,25,26].

This ELISA method provides a simple procedure to determine IC_50_s for anti-malarial drugs *in vitro* using *P. berghei*. Since studies focusing on the identification of novel drugs often involve the testing of the *in vivo* efficacy of drug candidates in small animal (rodent) models of malaria, the availability of simple assays to determine the *in vitro* drug-sensitivity of rodent parasites is useful. Specifically, this assay may help to determine whether a discrepancy between *in vitro P. falciparum* drug-sensitivity and *in vivo* rodent parasite drug-sensitivity is the result of intrinsic differences between the two parasite species or may be due to pharmacokinetic or pharmacodynamic characteristics of the drug in a live animal.

## Conclusion

In this study, the development of an ELISA-based *in vitro* drug assay for *P. berghei* is reported. This technique is easy to implement, fast (less than 3 hours), safe (avoids the use of radioisotopes) and economical (not requiring expensive equipment such as beta counters, flow cytometers or luminometers). Although the development of synchronized infections in mice is time consuming, this is a straightforward procedure which can be easily implemented in laboratories working with murine models. This *in vitro* assay represents a robust and useful alternative for the screening of new anti-malarial compounds in the mouse model of *P. berghei*.

## Abbreviations

WHO: World Health Organization; LDH: Lactate dehydrogenase; pLDH: Parasite Lactate dehydrogenase; ELISA: Enzyme-linked immunosorbent assay; IP: Intraperitoneally; PbA: *Plasmodium berghei* ANKA, PbAGFP, *Plasmodium berghei* ANKA GFP; MR4: Malaria Research and Reference Reagent Resource Center; NIH: National Institutes of Health; PBS: Phosphate buffered saline; RPMI: Roswell Park Memorial Institute; IV: Intravenously; OD: Optical density; IC_50_: Half maximal inhibitory concentration; SD: Standard deviation; HABA: 4'-hydroxyazobenzene-2-carboxylic acid; DELI: Double-site enzyme-linked lactate dehydrogenase immunodetection assay; BSA: Bovine serum albumin; TMB: 3,3',5,5'-tetramethybenzidine; unRBC: Uninfected red blood cell; cRMPI: Complete RMPI media.

## Competing interests

Authors do not have any competing interests.

## Authors’ contributions

The work was carried out in collaboration between all authors. POS and LJMC designed experiments. POS carried out the laboratory experiments, analysed the data, interpreted the results and wrote the manuscript. ED assisted in FACS experiments and FCS Express software analyses. LJMC conceived the study, interpreted results and revised the manuscript. JAF and JN discussed the results and revised the manuscript. All authors have approved the final manuscript.
